# Supplementary material for: Population size changes and selection drive patterns of parallel evolution in a host–virus system
Source: Nat Commun. 2018 Apr 27;9:1706. doi: 10.1038/s41467-018-03990-7 (PMC5923231; doi:10.1038/s41467-018-03990-7)
Supplement: Supplementary file 1 — Supplementary Information [file 41467_2018_3990_MOESM1_ESM.pdf]

Supplementary information

Population size changes and selection drive patterns of parallel evolution in a host-virus system

Frickel et al.

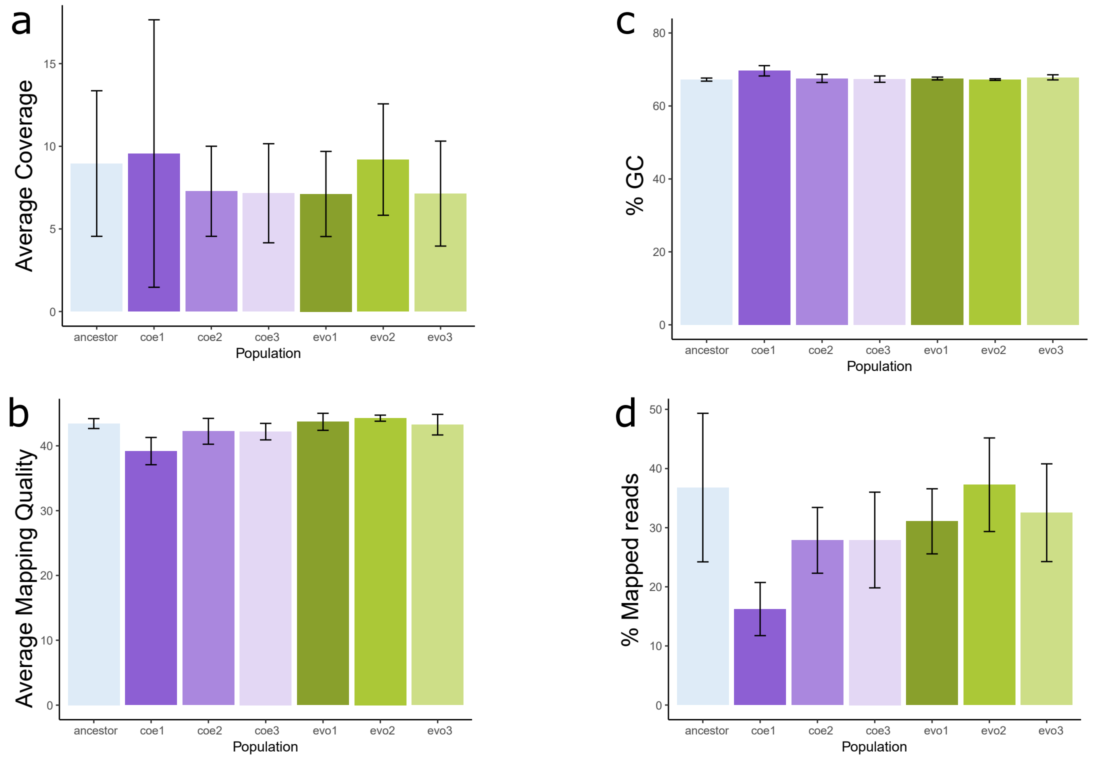

Supplementary Fig. 1. Quality statistics for sequencing data. (a) The average coverage over the whole genome was calculated for every clone. (b) The average mapping quality for all reads was calculated. (c) Average GC content for all mapped reads. (d) Percentage of mapped reads per clone. Bar plots represent the average per population. For all bars, error bars indicate standard deviations for n =10 clones. Anc = ancestor population, Coe = coevolved populations, Evo = evolved populations. Evolved populations are represented in green, coevolved populations are represented in purple and the ancestor population in light blue. The shade of the color corresponds to the different replicate populations.

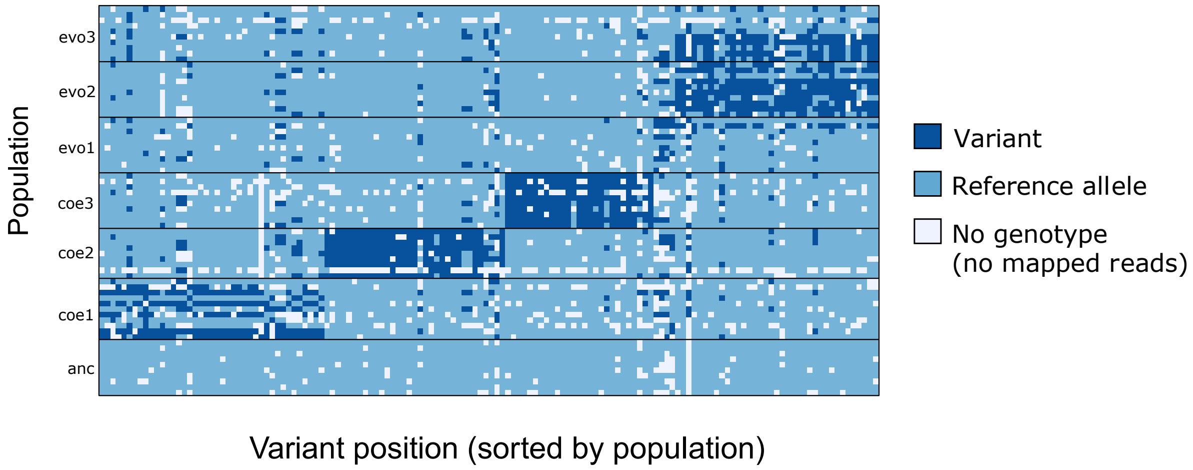

Supplementary Fig. 2. Frequency and distribution of high frequency variants. Each column represents a variant at a genomic position that contained variants at relatively high frequencies (≥0.5) in one or more of the sequenced populations (see main text). White color indicates no genotype could be called for this clone at this position due to no reads mapped. Light blue indicates that this particular clone had the reference allele at this location, dark blue indicates that this clone had an alternative allele at this position. Total amount of variants = 143. Variants are the same as represented (as frequencies) in Fig. 4. Abbreviations coe1 – coe3 correspond to the 3 coevolved populations. Abbreviations evo1 – evo3 correspond to the 3 evolved populations.

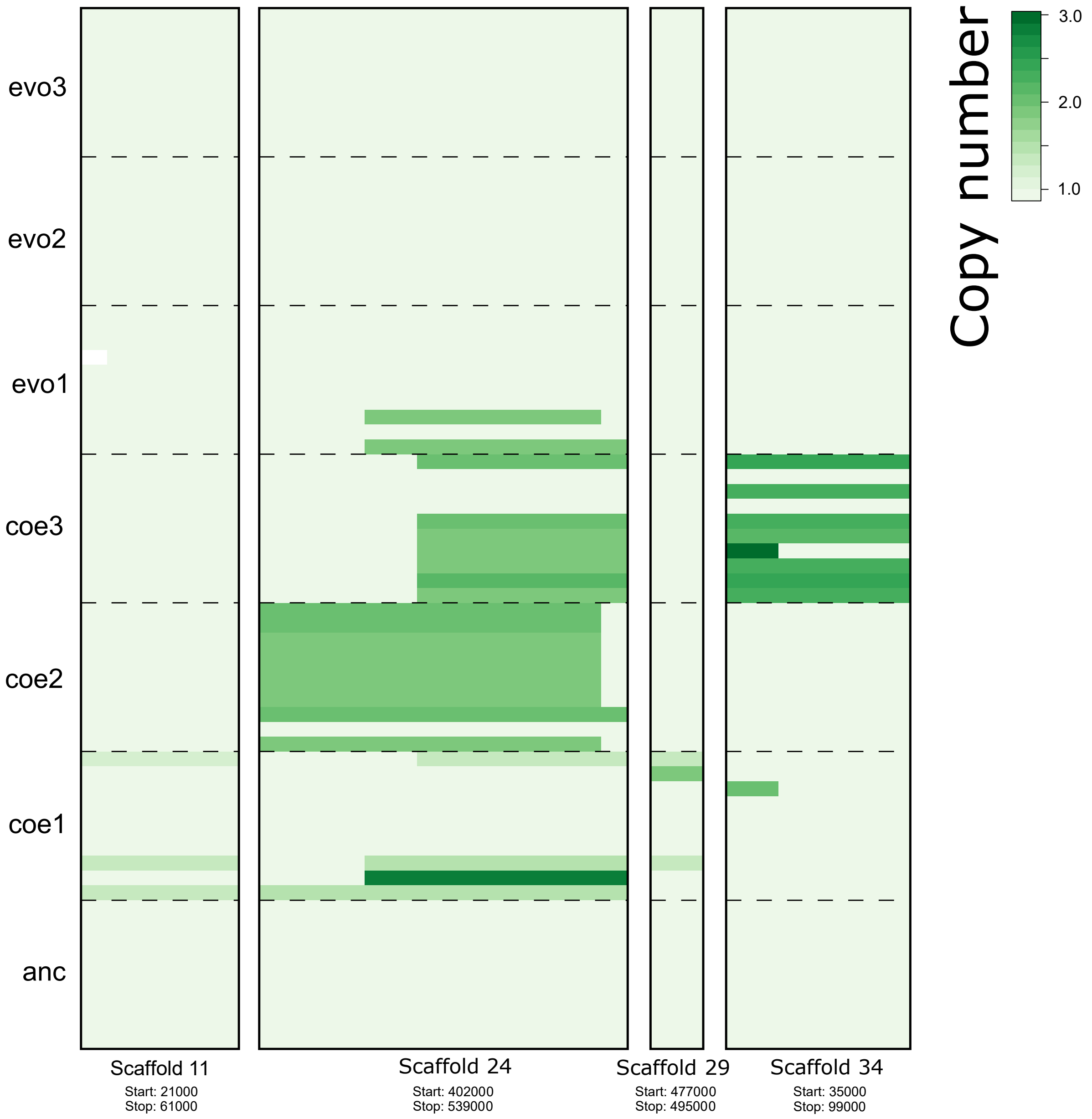

Supplementary Fig. 3. Copy number variants. All positions that had - on the population level - significant increase in copy number compared to the ancestor population. Color intensity represents the difference in copy number. Abbreviations coe1 – coe3 correspond to the 3 coevolved populations. Abbreviations evo1 – evo3 correspond to the 3 evolved populations and anc is the ancestor population.

Supplementary Table 1. General annotation of high frequency variants and genes in the shared duplicated genomic region. The general functions of genes containing high frequency variants in coevolved populations, evolved and both coevolved and evolved (shared) populations were annotated. The distribution of the genes within the different general processes is shown. Also the general functions of genes contained within the large genomic duplication were annotated.

	General Process	Fraction	Number of genes
**Coevolved**	Cellular Processes and Signaling	0.11	1
	Information Storage and Processing	0.11	1
	Metabolism	0.78	6
	Poorly Characterized	-	*6*
**Evolved**	Cellular Processes and Signaling	0.63	5
	Information Storage and Processing	0.13	1
	Metabolism	0.25	2
	Poorly Characterized	-	*1*
**Shared**	Cellular Processes and Signaling	0.33	1
	Information Storage and Processing	0.33	1
	Metabolism	0.33	1
	Poorly Characterized	-	*1*
**Duplication**	Cellular Processes and Signaling	0.36	4
	Information Storage and Processing	0.27	3
	Metabolism	0.36	4
	Poorly Characterized	-	*6*

Supplementary Table 2. Functional annotation of variants at high frequency and of genes in the duplicated genomic region (Fig. 6). Coe or Evo correspond to genes containing high- or moderate impact variants in coevolved (Coe) and evolved populations (Evo) or both (Coe & Evo). Dupl; are genes contained within the duplicated genomic region. Each of the genes was annotated with KOG id and the corresponding general process, functional description and KOG name.

Coe, Evo or Dupl	Gene	KOG Id	General Process	Function description	KOG Name
Coe	136424	KOG3365	Metabolism	Energy production and conversion	NADH:ubiquinone oxidoreductase, NDUFA5/B13 subunit
Coe	136223	KOG1310	Poorly Characterized	General function prediction only	WD40 repeat protein
Coe	134690				
Coe	13183	KOG0192	Cellular Processes and Signaling	Signal transduction mechanisms	Tyrosine kinase specific for activated (GTP-bound) p21cdc42Hs
Coe	139542	KOG0926	Information Storage and Processing	RNA processing and modification	DEAH-box RNA helicase
Coe	140110				
Coe	139079				
Coe	56489	KOG1046	Metabolism	Amino acid transport and metabolism	Puromycin-sensitive aminopeptidase and related aminopeptidases
Coe	144138				
Coe	143182	KOG2495	Metabolism	Energy production and conversion	NADH-dehydrogenase (ubiquinone)
Coe	37518	KOG0205	Metabolism	Inorganic ion transport and metabolism	Plasma membrane H+-transporting ATPase
Coe	53640	KOG3627	Metabolism	Amino acid transport and metabolism	Trypsin
Coe	56526	KOG2043	Metabolism	Cell cycle control, cell division, chromosome partitioning	Signaling protein SWIFT and related BRCT domain proteins
Coe & Evo	145076				
Coe & Evo	143599	KOG3544	Cellular Processes and Signalling	Extracellular structures	Collagens (type IV and type XIII), and related proteins
Coe & Evo	57723	KOG1202	Metabolism	Lipid transport and metabolism	Animal-type fatty acid synthase and related proteins
Coe & Evo	58355	KOG0979	Information Storage and Processing	Chromatin structure and dynamics	Structural maintenance of chromosome protein SMC5/Spr18, SMC superfamily
Evo	36848	KOG0192	Cellular Processes and Signalling	Signal transduction mechanisms	Tyrosine kinase specific for activated (GTP-bound) p21cdc42Hs
Evo	24868	KOG2835	Metabolism	Nucleotide transport and metabolism	Phosphoribosylamidoimidazole-succinocarboxamide synthase
Evo	137360				
Evo	32275	KOG2004	Cellular Processes and Signalling	Posttranslational modification, protein turnover, chaperones	Mitochondrial ATP-dependent protease PIM1/LON
Evo	140461	KOG3227	Information Storage and Processing	Transcription	Calcium-responsive transcription coactivator
Evo	140031	KOG3525	Cellular Processes and Signalling	Posttranslational modification, protein turnover, chaperones	Subtilisin-like proprotein convertase
Evo	135546	KOG3599	Metabolism	Inorganic ion transport and metabolism	Ca2+-modulated nonselective cation channel polycystin
Dupl	139351	KOG1474	Information Storage and Processing	Transcription	Transcription initiation factor TFIID, subunit BDF1 and related bromodomain proteins
Dupl	32691	KOG1077	Cellular Processes and Signaling	Intracellular trafficking, secretion, and vesicular transport	Vesicle coat complex AP-2, alpha subunit
Dupl	139355	KOG1740	Information Storage and Processing	Translation, ribosomal structure and biogenesis	Predicted mitochondrial/chloroplast ribosomal protein S17
Dupl	59012	KOG1889	Metabolism	Lipid transport and metabolism	Putative phosphoinositide phosphatase
Dupl	139357	KOG4660	Metabolism	Cell cycle control, cell division, chromosome partitioning	Protein Mei2, essential for commitment to meiosis, and related proteins
Dupl	139358	KOG1416	Information Storage and Processing	Translation, ribosomal structure and biogenesis	tRNA(1-methyladenosine) methyltransferase, subunit GCD10
Dupl	27124	KOG2708	Cellular Processes and Signaling	Posttranslational modification, protein turnover, chaperones	Predicted metalloprotease with chaperone activity (RNAse H/HSP70 fold)
Dupl	32696	KOG1164	Cellular Processes and Signaling	Signal transduction mechanisms	Casein kinase (serine/threonine/tyrosine protein kinase)
Dupl	32698	KOG0927	Poorly Characterized	General function prediction only	Predicted transporter (ABC superfamily)
Dupl	59016				
Dupl	59017				
Dupl	32701	KOG1444	Metabolism	Carbohydrate transport and metabolism	Nucleotide-sugar transporter VRG4/SQV-7
Dupl	139367				
Dupl	27165	KOG3274	Poorly Characterized	Function unknown	Uncharacterized conserved protein, AMMECR1
Dupl	59772	KOG0192	Cellular Processes and Signaling	Signal transduction mechanisms	Tyrosine kinase specific for activated (GTP-bound) p21cdc42Hs
Dupl	59021				
Dupl	37029	KOG2436	Metabolism	Amino acid transport and metabolism	Acetylglutamate kinase/acetylglutamate synthase

Supplementary Table 3. GO enrichment analysis of derived variants. Genes affected by high- and moderate impact variants (all derived variants) were used to perform GO enrichment analysis. Hits are the number of genes corresponding to the specific GO term, and Score is the associated p-value for the enrichment. GO enrichment was performed for Biological Process and Cellular Components.

Enrichment using ontology databases (JGI annotation)					
**COEVOLVED POPULATIONS**			**EVOLVED POPULATIONS**		
	**Hits**	**Score**		**Hits**	**Score**
Biological Process			Biological Process		
cellular nitrogen compound biosynthetic process	17	0.01337	purine nucleotide biosynthetic process	5	0.001419
nucleotide biosynthetic process	7	0.02952	purine nucleotide metabolic process	5	0.001419
branched chain family amino acid biosynthetic process	2	0.03396	nucleotide biosynthetic process	6	0.00147
amine metabolic process	14	0.04597	"nucleobase, nucleoside, nucleotide and nucleic acid metabolic process"	17	0.003185
cellular amino acid and derivative metabolic process	14	0.04597	"nucleobase, nucleoside and nucleotide biosynthetic process"	6	0.003865
cellular amino acid biosynthetic process	7	0.046229	"nucleobase, nucleoside, nucleotide and nucleic acid biosynthetic process"	6	0.003865
			nucleoside phosphate metabolic process	6	0.004209
			nucleotide metabolic process	6	0.004209
			purine ribonucleotide biosynthetic process	4	0.005486
			purine ribonucleotide metabolic process	4	0.005486
			ribonucleotide biosynthetic process	4	0.005486
			ribonucleotide metabolic process	4	0.005486
			proteolysis	10	0.008892
			cellular nitrogen compound metabolic process	20	0.010868
			"nucleobase, nucleoside and nucleotide metabolic process"	6	0.011722
			nucleoside monophosphate biosynthetic process	3	0.014284
			nucleoside monophosphate metabolic process	3	0.014284
			nitrogen compound metabolic process	20	0.014438
			aromatic amino acid family catabolic process	1	0.026279
			aromatic compound catabolic process	1	0.026279
			L-phenylalanine catabolic process	1	0.026279
			L-phenylalanine metabolic process	1	0.026279
			regulation of ARF protein signal transduction	1	0.026279
			regulation of Ras protein signal transduction	1	0.026279
			regulation of signaling pathway	1	0.026279
			regulation of small GTPase mediated signal transduction	1	0.026279
			purine nucleoside monophosphate biosynthetic process	2	0.032055
			purine nucleoside monophosphate metabolic process	2	0.032055
			purine ribonucleoside monophosphate biosynthetic process	2	0.032055
			purine ribonucleoside monophosphate metabolic process	2	0.032055
			ribonucleoside monophosphate biosynthetic process	2	0.032055
			ribonucleoside monophosphate metabolic process	2	0.032055
Cellular Component			Cellular Component		
integral to membrane	15	0.021418	biotin carboxylase complex	1	0.021664
intrinsic to membrane	15	0.021418	ribonucleoside-diphosphate reductase complex	1	0.021664
endoplasmic reticulum	3	0.026039	chromosome	2	0.034973
cytoplasm	11	0.033591	protein complex	10	0.039957
			mitochondrial outer membrane	1	0.042877
			organelle outer membrane	1	0.042877
			phenylalanine-tRNA ligase complex	1	0.042877
			phosphoribosylaminoimidazole carboxylase complex	1	0.042877
			protein phosphatase type 2A complex	1	0.042877

Supplementary Table 4. GO enrichment analysis of genes in genomic duplication (Fig. 6). Genes contained within the large genomic duplication were used in GO enrichment analysis. Hits are the number of genes corresponding to the specific GO term, and Score is the associated p-value for the enrichment. GO enrichment was done for Biological Process and Cellular Components.

Enrichment using ontology databases (JGI annotation)		
**DUPLICATION**		
	**Hits**	**Score**
Biological Process		
proline biosynthetic process	1	0.011027
regulation of translational initiation	1	0.011027
arginine biosynthetic process	1	0.013768
posttranscriptional regulation of gene expression	1	0.013768
proline metabolic process	1	0.013768
regulation of translation	1	0.013768
arginine metabolic process	1	0.016502
protein amino acid phosphorylation	2	0.027959
glutamine family amino acid biosynthetic process	1	0.032777
phosphorylation	2	0.033439
glutamine family amino acid metabolic process	1	0.043501
Cellular Component		
AP-type membrane coat adaptor complex	1	0.010372
clathrin adaptor complex	1	0.010372
membrane coat	1	0.048626
